# Teaching Medical Procedural Skills for Performance

**DOI:** 10.3390/clinpract14030067

**Published:** 2024-05-16

**Authors:** Kersi Taraporewalla, Paul Barach, André van Zundert

**Affiliations:** 1 Faculty of Medicine, The University of Queensland, Brisbane, QLD 4072, Australia; 2 Department of Surgery, Imperial College London, London SW7 2AZ, UK; 3 School of Medicine, Sigmund Freud University, 1020 Wien, Austria; 4 School of Population Health, Thomas Jefferson University, Philadelphia, PA 19144, USA; 5 Department of Anaesthesia and Perioperative Medicine, Royal Brisbane and Women’s Hospital, The University of Queensland, Brisbane, QLD 4029, Australia

**Keywords:** procedural skills, training, performance, constraint-led approach, competence, medical, patient safety

## Abstract

Procedures are a core element of medical professional practice. Today’s training approach was formulated in the mid-twentieth century based on a computer analogue of the brain. Despite minor modifications, the system has remained relatively unchanged for the past 70 years. It delivers competence. However, competence is not reliable performance. The inability to adapt to the variety of patients and variations in the performance environments, such as the operating room, results in patient morbidity and mortality. There is a need for changes in the development and training of medical procedural skills based on current theories of skill acquisition, movement theory, and motor control. Achieving optimal performance necessitates the ability to adapt through training in diverse patient and performance environments rather than merely imitating prescribed movements. We propose a novel model of training, the Constraints-Led Approach, which allows for robust training by altering the factors affecting skill acquisition and lifelong learning.

## 1. Introduction

Society demands basic competence standard from medical professionals, while individual patients desire safe, harm-free expert care [1]. Medicine is inherently high risk, as it is prone to significant complications that can harm patients [2]. Healthcare professionals and health authorities invest substantial efforts into delivering high-quality medicine and uphold the fundamental principle of ‘do no harm’ [3].

Technical skills are vital for the performance and quality of patient care. While non technical skills are key to safe care, technical skills, [4] in fields such as surgery, may be more important for a successful outcome [5]. For instance, Birkmeyer et al. discovered significant variability in the technical skills among practising bariatric surgeons. They found that surgeons with superior surgical skills working high volume systems experienced fewer postoperative complications and lower rates of reoperation, readmission, and emergency department visits [6].

Failure to perform a task appropriate to the contextual requirements can result in patient morbidity and mortality. In a study conducted in the US on surgical malpractice claims, technical errors accounted for 133 cases of the 258 claims involving serious injuries, representing 49% of patients with permanent disability and 16% of deaths [7]. Further, in a retrospective failure analysis of laparoscopic hernia repair, video recordings revealed technical errors contributing to the observed complications [8].

Technical failure is also common in anaesthesia. Failure to perform peripheral intravenous cannulation on the first attempt is reported in 40% of cases [9]. In central venous access procedures, mechanical complications are estimated in 5 to 19% of cannulation attempts with arterial puncture occurring in 4.2% to 9.3% of cases [10]. Additionally, guidewire retention is increasing, with operator error by trainee doctors responsible for 88% of the cases [11].

Ready reported an overall failure rate of 32% in thoracic epidural catheter insertions in a large teaching institution in 1999. Among these failed catheters, 11% were not in the epidural space when tested with a local anaesthesia bolus [12]. In 2016, the failure rate for thoracic epidural catheters was 24%. Difficulties in teaching thoracic epidural insertion were cited as part of the problem and the reason for the lack of improvement [13]. In 2.9% of cases, operators could not adequately localise the thoracic epidural space [14].

There are numerous factors contributing to technical errors in medicine. In surgery, these encompass organisational dynamics, surgical and operating theatre teamwork, patient-related pressures, patient factors, and personal stress experienced by the surgeon [15]. The patient characteristics include the ASA grade, age, BMI, and surgical pathology [15]. Surgeon-related factors include fatigue, sleep deprivation, illnesses and family problems [15]. From a complex system’s perspective, these factors are considered system constraints and can be categorised as environmental, performer, and task-related [16]. Given that many technical errors and complications are reported in patients with pathophysiological variations or challenging situations, it is imperative to examine if current procedural skill teaching adequately trains practitioners to cope with real-world demands at all levels.

## 2. Contemporary Medical Procedural Skill Training

Movement theorists in the mid-twentieth century relied on a cybernetic model of the human brain for generating movement [17]. They proposed that the brain controls all human actions through motor plans or schemas [18]. Once a goal is set, such as performing intravenous cannulation, a stored representation of the task is activated. The schema is acquired through cognitive learning and developed through practice and experience. The brain activates the relevant schema to perceive relevant information from the environment pertaining to the goal. This perceived information is linked with a general motor plan with adjustable parameters. Following parameter adjustment, activation of the motor plan leads to a sequence of movements regulated by the brain via the motor cortex, cerebellum and other neuro-motor structures. Movement control of the action occurs through feedback loops within the brain [19]. The schema must be developed and stored prior to practice and performance.

Taxonomies of psychomotor learning were invoked to formulate the teaching approach. The widely referenced 1972 Simpson taxonomy posits that motor learning develops in stages: perception (awareness of performance), set (ready to perform), guided response, mechanism (developing routine actions), complex overt response (demonstrating proficiency), and adaption [20]. Procedural skills are best learned in a stepped, sequential teaching approach, beginning with the cognitive development of the motor plan [21]. In 1997, Kovacs produced a four-stepped method of ‘learn, see, practice, and do’, emphasising the demonstration and measurement of competence [20]. This model requires learners to develop a mental representation of the task by memorisation of the movement steps followed by visualisation and verbal descriptions in the next sequential step. Practice was focused on replicating the actions and movements with immediate feedback correction. Learning was measured by examining performance on a standardised task with competence measured by validated checklists. This representative model of procedures and its teaching with minor modifications has persisted over time. In 2015, it was reinforced by the ‘learn, see, practice, prove, do, maintain’ framework following a review of the medical literature [22]. This approach requires a different training method for developing expertise once the skill is acquired.

Several limitations of the competence-based representational motor learning model may explain the technical errors observed in studies. They include the reliance on a computer analogue model of the brain, how humans produce movements and competence-based teaching.

### 2.1. Problems with the Computer Analogue Model of the Brain

Humans are not computers with programmed motor plans. Suppose movements solely result from executing a stored motor plan. In that case, we would expect each motor plan execution to produce the same result each time it is performed in the same environment. This is not the case. If asked to draw five circles 2.5 cm in diameter that are all the same, you will notice that each one is different. Similarly, when we walk, the pace, stride length, and direction continuously vary with the circumstances and location. In clinical practice, movements made during intravenous cannulation or endotracheal intubation adapt to the nature of the patient, the location, and circumstances, amongst other factors. This dynamic nature of human movement underscores the limitations of motor plans and the training approach dependent on this theory.

The vast repertoire of life’s movements and the associated parameters required for each action present a storage problem for the human brain: where are the motor plans for the movements stored? There would need to be an infinite storage capacity in the nervous system to contain all the plans necessary for the variety of movements available [23]. In other life forms, such as ants, bees, insects and octopi, complicated actions are generated with few neurons, suggesting motor plans may not be stored if they do exist.

Motor equivalence poses another challenge [23]. For instance, lifting a cup of tea for a drink can be accomplished using various coordination patterns. The upper limb could do most of the lifting, or the lifting action could be distributed between the upper limb and the trunk. The theory of a general motor plan with movement parameters such as speed and direction fails to elucidate how a coordination pattern is chosen or which muscles will be activated in each situation [23]. In the clinical setting, this problem relates to storing many motor plans for medical tasks performed in varied performance and environmental contexts.

A motor plan alone cannot account for the sudden adjustment required when the movements are perturbed. For example, while lifting a cup of tea from the table, a disturbance to the direction of the arm by someone bumping the arm, the body automatically readjusts the movement to achieve the desired outcome. Similarly, in the clinical setting, surgeons suddenly change how they manipulate a laparoscope based on the view they obtain and their physical constraints in the operating theatre. Moreover, novel movements required in unique circumstances are made without prior learning or representation development, which traditional motor plan theory does not fully explain [23].

Movement is a dynamic entity that emerges due to continuous interactions in the performer–environment system, within the performer’s limitations, environment, and task, to satisfy the intentions or goals [24]. This ecological–dynamic movement theory explains why experts can state their aims and objectives which might be quite different than how they move in different circumstances [25].

### 2.2. Problems with the Representative Model of Psychomotor Skills

The representative model of psychomotor skills aims to form a mental schema of the psychomotor task. This process typically begins with a verbal description of the actions. This is stored in memory. The model is then progressively developed through further explanation and demonstration (verbal and visual). The model is the foundation for executing actions. External feedback and practice refine the model to reduce conscious, deliberate attention to movement and automate them. Expertise develops with deliberate practice aimed at improving the representation. The Peyton and Walker four-step approach is commonly used to create the mental model of the medical task. It consists of demonstration, deconstruction, comprehension, and practice with feedback [26]. This approach assumes a unique way to perform the task in all circumstances.

The representative model assumes cognitive control of movements, which can result in an extensive cognitive workload for complex or complicated medical tasks. This approach carries the risks of cognitive incongruence during the demonstration of the steps. Signal noise is not tolerated in this training approach. The learner needs to duplicate the prescribed steps without variation. The approach encounters challenges with unusual variations. A patient with unusual anatomy, such as scoliosis, may pose a significant problem with an increased risk of dural puncture or catheter misplacement if a standard approach for epidural placement is followed.

The representative model overlooks individual variability amongst learners, such as physical size, prior experience and skill level. For instance, a person of 55 kg cannot adequately produce the 5 cm depth of chest compression for the required duration in cardiopulmonary resuscitation in the same manner as a resuscitator of 80 or 100 kg. Different muscles and body parts are engaged to accomplish the same task, highlighting the importance of considering individual differences in skill acquisition and implementation.

Assuming a correct fixed way to perform a specific task can become problematic as human movements are not repeatable and inherently variable, being influenced by factors such as the patient, location, and the context of care delivered. In less commonly practised tasks, such as when doing a stellate ganglion block or coeliac plexus block, establishing the “correct” directions becomes difficult. Such difficulties are noted in teaching thoracic epidurals [13].

Representative models are often incomplete and oversimplify the coordination required to perform tasks. The brain must coordinate various bodily systems during movements, including the cardiovascular, endocrine, neuromuscular, sensory, and joint systems. Representative models fall short in describing how the coordination of the many systems develops and is controlled. In contrast, viewing humans as complex neuro-biological systems capable of self-organisation and pattern forming with the emergence of coordination removes the necessity for a rigid representation. The performer needs to set goals and intentions. Skill teaching requires learners to be educated in the intentions of movements as part of the perception–movement complex [27].

The representative model is static and lacks the flexibility to adapt to environmental variations and dynamic changes that can occur during task performance. Representative models need more nuances for accurate execution in unforeseen circumstances and variations. They potentially inhibit the development of adaptability and critical thinking [27]. For instance, a cognitive-based representation model of performing an epidural in labour does not account for scoliosis, moving patients in pain, and a deformed spinal column. This may explain the difficulties trainees have with such patients. This underscores the need to teach skills for effective performance to manage the diversity encountered in life.

Representative models assume linear learning, and the role of practice is to eliminate errors and produce proportionate improvements in performance (continuous quality improvement). This is not observed in practice. Some learners may experience regression with more training. Many trainees have experienced a sudden succession of failures with intravenous cannulation after being successful during training. Learning is a complex phenomenon characterised by skips and jumps [28]. Further, as trainees advance in perceptual–motor learning, the way they process the task and action qualitatively changes through different their phases of development, [29], challenging the nature of a representative model.

In summary, a representative model relies on cognitive learning, assumes a fixed method of performance, neglects environment and patient variations, and lacks the ability to adapt to variations encountered in life.

### 2.3. Problems with the Competence-Based Model of Training

The assessment of competence is a recognised key component of the representative model [17,20,21,22]. The methods for the measurement and assessment of competence vary between authors. This includes the number of cases performed, time to perform the learned task, direct observations, structured knowledge tests and checklists. Assessors emphasise the importance of objective competence assessments and that the measures are reliable, valid and homogeneous [30]. The cases are often standardised, and fixed checklists are used [30]. A systemic review of the competence assessment of medical studies determined that there are no robust tools explicitly designed to assess the competence of procedural skills for a high stakes certification examination [30]. This assessment method can significantly affect teaching and learning. Teachers may focus on teaching perfect movements in the fixed, set cases, while learners prioritise performing well for assessment, creating difficulties with transfer to various clinical cases. The reductionist approach to measuring competence tends to overlook the integration of components of movements and the interrelationships between them.

Competence is not the same as performance. The assertion of competence in a simulated setting does not guarantee the transferability of the skill to the clinical workplace [30]. Competence typically refers to the ability to perform in the idealised conditions of the teaching and testing environment. However, patients want the medical practitioner to perform procedures for their individual specific physiological, pathological and body conditions in the specific performance environments. A competence measuring tool tends to have a checklist for selected circumstances. A performance measuring tool is required to account for the diversity and adaptability of performance requirements.

While the representative model has been valuable in medical procedural skill development, it overlooks the nuances of accurate execution in unforeseen circumstances and the variations present under real world conditions. As reported above, this may lead to difficulties in skilled performance. What may be needed is an approach to procedural skill training that delivers reliable performance by encouraging adaptability and agility in approaches.

## 3. The Constraint-Led Approach to Developing Medical Psychomotor Skills

The Constraint-Led Approach (CLA) offers an alternate method that utilises the ecological–dynamic concept of goal-directed movements. It has been highly successful and accepted in the sports domain [31]. Goal-directed movements emerge from dynamic interactions between the performer and the environment. It requires learners to be educated in intentions that satisfy the goal and become attuned to the critical variables in the environment that regulate movements. They calibrate their actions based on the limitations of the environment, task requirements, and individuals. Rather than prescribing fixed movements, each learner develops their individual movement solution to the problem based on their specific and evolving circumstances.

Instructors create environments that allow learners to develop the required movements by adjusting the constraints (limitations within the learner, environment and task) or enhancing perception. They also provide performance environments rather than teaching environments. The active involvement of instructors during training is crucial, as they strike a balance between stability and variability and introduce signal noise to allow for adaptation while keeping the patient safe. Instructors can improve the perception of critical elements by varying the training environment to reflect the various performance environments the trainee will encounter including adjusting for the critical elements regulating actions, decision-making within the task, and adaptability. This develops performance rather than competence.

CLA considers learning to be nonlinear. It uses the principles of nonlinear pedagogy, which overlap with the principles of the constraint-led approach. They include representative learning design, task simplification (not deconstruction), constraints (limitations), manipulation, attentional focus, to produce functional variability [32]. The CLA approach is integrated, linking movements to the theoretical concepts considered necessary in the representative model. It uses direct learning, thereby reducing the cognitive load. This approach does not preclude developing skills in classrooms, simulation or clinical environments. The advantages of CLA include promoting direct learning, fostering adaptability, and reducing learner’s cognitive workload. Studies have shown that CLA can effectively be used to teach procedural tasks and is more efficient in skill acquisition than the traditional approach in performing effective external chest compressions [33].

The Constraints-Led Approach (CLA) does not dismiss the importance of practice, simulation-based learning, using guidelines and protocols, or breaking down complex procedures into smaller components for teaching purposes. Instead, it clarifies how these teaching and learning tools could be effectively utilised. Practice remains essential in skill acquisition, but CLA emphasises the importance of striking a balance between stability and variability to clarify the components of appropriate movements. Simulation-based learning offers a safe environment for learners to practice, thus minimising patient harm. Simulation must replicate representative performance environments and integrate both practical and cognitive components seamlessly rather than presenting in isolation or sequentially. Guidelines and protocols play a valuable role in education by defining the algorithms for performing procedures. They serve as tools for teaching intentions and goals, providing learners with a structured framework. Ultimately, CLA underscores the importance of utilising various teaching and learning tools in a complementary manner to optimise skill acquisition performance in medical education.

CLA can also be utilised to enhance procedural performance in medical settings. For example, in the case of acute ischemic stroke management, despite existing guidelines and protocols, there may still be challenges in achieving the optimal administration rates of intravenous tissue plasminogen activator (tPA) in emergency departments [34]. By adopting CLA principles, such as analysing environmental, task, and performer constraints, healthcare providers can identify areas for improvement and adjust these constraints to enhance performance. This may involve restructuring acute ischemic stroke management care flow processes to meet recommended time targets, which has been shown to improve patient outcomes and response times. Simulations can be instrumental in training healthcare providers to adapt to these adjusted constraints effectively, allowing for experimentation and learning with minimal patient harm. Healthcare systems can use CLA to evolve and improve, ultimately leading to better outcomes for patients presenting with acute ischemic stroke and other medical conditions.

Several authors have highlighted the benefits of CLA based approaches in teaching clinical tasks. Tjiam et al. published an approach to adaptation by introducing progressively complex real cases in a simulator-based course to improve performing a nephrostomy [35]. Unalan et al. developed basic arthroscopic skills via an integrated system that translated to clinical knee arthroscopic procedures [36]. Using a constraints-led approach (CLA), Renden and Dikken taught nurses to wash their hands without error in busy ward environments [37].

## 4. Conclusions

Modern medicine demands appropriate and safe performance by adapting to existing circumstances rather than competence in limited learning situations. While the cognitive-based representative model has served well over the past decades, the medical procedural skill training approach needs to evolve to methods that better align with the complexities and uncertainties of real-world clinical practice. Developing performance rather than competence standards may lead to fewer technical errors and reduced morbidity and mortality, benefiting patients and healthcare providers.
